# Colony stimulating factor-1 and leukemia inhibitor factor expression from current-cycle cannula isolated endometrial cells are associated with increased endometrial receptivity and pregnancy

**DOI:** 10.1186/s12905-017-0418-7

**Published:** 2017-08-22

**Authors:** Felipe Camargo-Díaz, Valeria García, Azucena Ocampo-Bárcenas, Humberto González-Marquez, Esther López-Bayghen

**Affiliations:** 1 Laboratorio de Investigación y Diagnóstico Molecular, Instituto de Infertilidad y Genética SC, INGENES, Mexico City, Mexico; 2 Departamento de Toxicología, Centro de Investigación y de Estudios Avanzados del IPN, México City, CDMX Mexico; 3 Programa de Doctorado en Ciencias Biológicas y de la Salud, Universidad Autónoma Metropolitana, Unidad Iztapalapa, Mexico City, Mexico

**Keywords:** Endometrial receptivity, CSF, LIF, HOXA10

## Abstract

**Background:**

Poor endometrial quality is associated with more than a third of embryo implantation failures. Current ultrasonography technology lacks the capacity to determine efficiently the endometrial receptivity during ongoing cycle transfers. We analyzed the relationship between the gene expression profile associated with implantation and clinical pregnancy from endometrial cells taken during embryo transfer.

**Methods:**

Seventy-six patients submitted to a standard ovarian stimulation protocol, in vitro fertilization, and good quality embryos were collected (morphological assessment). Endometrial samples were taken with ultrasonography guidance and cells were Hematoxylin and Eosin stained for morphological identification. Total RNA was extracted and the expression of Mucin 1 (MUC1), Homeobox A10 (HOXA-10), Leukemia Inhibitor Factor (LIF), Colony Stimulating Factor-1 (CSF-1), and ribosomal 18 s (endogenous control) were analyzed using RT-qPCR. Presence of a gestational sac, β-hGC (≥10 mIU/mL on Day 20), and a fetal heartbeat were used to determine a positive embryo implantation and pregnancy.

**Results:**

Samples collected from same cycle embryo transfer showed clear morphological staining for endometrial cells (80–90% of the cells). Cells in the sample were molecularly identified as the endometrium (HOXA-10 positive and MUC-1 negative). CSF-1 expression was 4.55-fold and LIF expression was 12.25-fold higher in patients who became pregnant. Both increases were statistically significant (*p* < 0.05).

**Conclusions:**

Here, we provide evidence of a new method to assess endometrial receptivity. Furthermore, we demonstrate that the expression profile, based on LIF and CSF-1, showed a difference between a receptive and a non-receptive endometrium.

## Background

In assisted reproductive technology, pregnancy and birth rates following in vitro fertilization (IVF) remain low. Two out of three IVF cycles fail to result in pregnancy and more than eight out of ten transferred embryos fail to implant [1]. Failures are usually associated with inadequate oocyte or embryo quality, number of implanted embryos, and endometrial receptivity, which accounts for a majority of all implant failures. Current methods for assessing endometrial receptivity are limited to morphological and blood flow examinations; however, the predictive capability of this methods remains suboptimal. Yet, with the numerous biomarkers that can vary considerably between ethnicities, there remains an incomplete understanding of key genes associated with endometrial receptivity.

The endometrium is a highly dynamic tissue which undergoes physiological changes in response to steroid hormones. Ultimately, the endometrium must achieve a receptive status in a synchronized manner alongside the arrival of the implanting blastocyst during the window of implantation [2]. Implantation of the embryo into the endometrium is a critical event in establishing pregnancy. For a successful implantation, there must be developmental synchrony between the embryo and the endometrium [2, 3]. This was first observed in the 1960s during embryo transfer experiments in animals and has held true for humans. The human endometrium becomes receptive to implantation in each normal menstrual cycle following ovulation and is driven by rising concentrations of both estrogen and progesterone. This receptivity lasts for only about 4 days in the mid-secretory phase, typically between days 19 and 21 of menstrual cycle [2, 3].

Many genes and proteins have been identified to correlate with endometrial receptivity. For example, Homeobox A10 (HOXA-10) transcription levels significantly decreased in infertile patients, when compared to controls [4]. Mucin 1 (MUC1) expression was significantly reduced 7 days after the luteinizing hormone peak in glandular epithelial cells and the endometrial lumen in women that suffer from recurrent pregnancy lost [5]. Colony Stimulating Factor 1 (CSF-1) was down-regulated in the endometrium from patients with recurrent miscarriages [6]; moreover, increased serum CSF-1 levels were associated with augmented pregnancy rates. Furthermore, in women undergoing IVF, CSF-1 serum levels increased throughout stimulation until the day of oocyte retrieval and then decreased until embryo transfer [7]. Lastly, numerous reports have suggested the link between Leukemia Inhibitory Factor (LIF) expression and endometrial receptivity [8–11]. LIF mRNA is expressed in the endometrium of normal fertile women but is significantly decreased in infertile women. In uterine flushing samples, LIF was undetectable in 88% of infertile women [12]. LIF concentrations were 15 times higher in patients considered to have a receptive endometrium by the Endometrial Receptivity Array [2]. This would suggest that the expression pattern of key genes, such as HOXA-10, MUC1, CSF-1, and LIF expression profile could indicate endometrial receptivity.

A striking limitation for gene expression studies has been the need to use cells collected by endometrial biopsy for profiling [13]. Sequential invasive sampling of the endometrium during a single cycle introduces confounding changes related to wounding and can alter the candidate biomarkers [14]. More importantly, the approach used to sample the endometrium, such as biopsying the anterior and posterior walls of uterus during the window of implantation, is incompatible with clinical use in an active cycle and therefore does not enable the direct association of most biomarkers with implantation rates and clinical outcome [15]. However, using the endometrial cells that result attached to the cannula used for embryo transfer, which directly touches the endometrium, could be a viable source to assess the endometrium’s receptivity. Thus, we conducted this study to assess the plausibility of endometrial cells collected during embryo implantation (Day 3), using a minimally invasive technique, to determine key factors associated with endometrial receptivity, namely CSF-1 and LIF.

## Methods

### In vitro fertilization and endometrial cell isolation

Seventy-six patients were selected for this cohort study from Mexico City, Mexico. All patients were subjected to controlled ovarian stimulation for 10 days. The controlled ovarian stimulation protocol consisted of administering a daily dose of a Gonadotrophin-releasing hormone agonists and antagonists [0.25 mg/day Cetrorelix, Cetrotide (Merck, Darmstadt, Germany) or 0.25 mg/day Ganirelix acetate (Orgalutran MSD, Kenilworth, NJ, USA)] in the luteal phase after menses. Gonadotropins were administered in variable doses with a minimal daily dose of 300 IU, depending on patient’s age and ovarian responsiveness with further adjustments according to serum estradiol (E2) levels and vaginal ultrasound measurements of follicular diameters obtained every 2 or 3 days. Stimulation was prolonged until the mean diameter of leading follicles was >18 mm. Recombinant human Urinary Chorionic Gonadotropin (Choragon 1000 IU, Laboratorio Ferring, Saint-Prex, Switzerland) was administered and oocyte retrieval was conducted 36 h after administration with ultrasound guidance. All 14–18 mm follicles were aspirated (typically 6 to 18), 6 to 14 oocytes were obtained with an average of 10.5 ± 2.5 oocytes per patient. Embryos obtained ranged from 1 to 10 per patient (fertilization rates were around 70%). An Embryologist monitored and recorded information about fertilization rates, embryo development, and embryo morphology for each oocyte. Embryo transfer was performed on Day 3 after an ultrasound confirmation of the uterus conformation as well as the evaluation of the endometrium for a tight trilaminar structure (7–11 mm). Once these conditions were identified, a flexible Wallace cannula is introduced through the cervix (uterine neck) ensuring contact with the endometrium with ultrasonography guidance. The cannula was removed and washed with PBS. The cells were collected with 200 μl of PBS to obtain a cellular suspension by rigorous agitation. 20 μl were taken to perform an endometrial imprint and the remaining 180 μl were recovered into a 1 ml Eppendorf tube containing the TRIzol® LS reagent and stored at −70 °C until RNA extraction.

### Pregnancy and allocation of patients

Positive pregnancy and implantation was assessed by plasma β-hGC concentration ≥ 10 mIU/mL on Day 20 and ongoing pregnancy as defined as the presence of gestation sac after 20 weeks and the number of positive heartbeats on ultrasound per embryo transferred. The patients were separated into two groups depending on the diagnosis of pregnancy.

### Cytological determination of endometrial cells

The endometrial cells were attached to a microscope slide and then stained with Hematoxilin-Eosin as follows: incubated with fixative agent for 1 min, followed by the Eosin dye for 1 min, washed with water and then exposed to Hematoxilin for 1 min, and a final wash with water. The samples were viewed with an Olympus BX50 microscope with an attached Optronics MagnaFire digital camera. The cells were deemed as endometrial cells as described elsewhere [16].

### RNA extraction

Total RNA was extracted from the cellular suspension using the TRIzol® LS Reagent (Ambion) according to manufacturer’s instructions. Briefly, all samples were mixed with 70% chloroform and incubated five minutes at room temperature, followed by centrifugation at 12,500 g for 15 min at 4 °C. The supernatant was transferred into a new Eppendorf tube containing 150 μl isopropanol. Samples were incubated for 10 min at room temperature and centrifuged at 12,500 g for 15 min at 4 °C. The pellet was washed with 100 μl of 75% ethanol and then centrifuged at 12,500 g for 5 min. The pellet was air-dry for 10 min. RNA was suspended in 0.1% DEPC water. All samples were analyzed by spectrophotometry to determine RNA concentration, yield, and purity (Epoch/Biotek, Winooski, VT, USA).

### Quantitative reverse transcription PCR (RT-qPCR)

RT-qPCR reactions were performed on the StepOnePlus apparatus (Applied Biosystems) using the Kappa Syberfast kit (KAPA Biosystems). DNA primers were designed and standardized to amplify MUC1, HOXA-10, CSF-1, LIF, and ribosomal S18 (endogenous control). Primers sequences are shown in Table 1. All reactions were quantified in duplicate. The reaction mixture consisted of 5 μl 2X KAPA SYBR® FAST RT-qPCR Master Mix (Woburn, MA, USA), 0.2 μl ROX, 0.2 μl dUTP (10 mM), 0.2 μl forward and reverse primers (20 pmol), 0.2 μl KAPA RT, 100 ng of RNA sample and DEPC water for a total volume of 10 μl. RT-qPCR conditions were 1 cycle of reverse-transcription at 42 °C for 5 min, 1 cycle of reverse-transcriptase inactivation at 95 °C for 5 min, 40 cycles of amplification at 95 °C for 15 s, 56 °C for 30 s, then 72 °C for 30 s. A melting curve was constructed after final amplification cycle. The relative abundance of each amplicon was calculated using the 2^-ΔΔCt^ method.

**Table 1:** qPCR Primers

Gene	Forward Primer	Reverse Primer	Size (bp)
18 s	5′-CGAAGATATGCTCATGTGGT-3′	5′-GACCTGGCTGTATTTTCCAT-3′	183
MUC-1	5′-TTTCCAGCCCGGGATACCTA-3′	5′-CTGGCCCTGAAGAACCTGAG-3′	250
HOXA-10	5′-GACAAATGCCCCAAAGTCTC-3′	5′-CTGAGAAAGGCGGAAGTAGC-3′	129
CSF-1	5′-GGAGACCTCGTGCCAAATTA-3′	5′-GGCCTTGTCATGCTCTTCAT-3′	223
LIF	5′-TGAACCAGATCAGGAGCCAACT-3′	5′-CCACATAGCTTGTCCAGGTTGTT-3′	127

All the PCR products were resolved through capillary electrophoresis using the BioAnalyzer Labchip GX (Caliper). Products showed a single band corresponding to the predicted base pair length. Moreover, the bands were cloned and analyzed, via sequencing, to verify their identity by direct cloning with the CloneJet system and sequenced using the BigDye system. Briefly, the amplicon fragments were purified using the GeneJet Gel Extraction kit (Fermentas) and ligated into a pJET1.2/blunt vector following the manufacturer’s protocol (Fermentas, ThermoFisher, Waltham, MA, USA). Plasmids were transformed into TOP10 competent bacteria and grown in LB medium (Ampicillin, Pisa SA Laboratorios Mexico 100 mg/ml) for 16 h at 37 °C. Plasmids were extracted from the bacteria using the QIAprep Spin Miniprep Kit (QIAGEN). The amplicon’s identity was verified by sequencing using BigDye Terminator v3.1 reagent and the RV primer 3 (3′-CTAGCAAAATAGGCTGTCCC-5′; Applied Biosystems, Foster City, CA, USA). Samples were sequenced with the ABI PRISM 3700 analyzer (Applied Biosystems) and aligned using Blast software.

### Statistical analysis

Data are expressed as the mean ± standard error. Student’s *t*-test was performed to determine whether there were significant differences between groups (Sigma Plot 12 Software).

## Results

### Description of study participants

Our cohort consisted of 76 women from central Mexico. A majority of the women suffered from primary infertility (59.2%) or secondary infertility (36.8%, Table 2). The leading cause of infertility was age (50.0%), followed by endometriosis and tubal factor (11.8%). Poor oocyte quality and fertilization failure only accounted for one case each (1.3%).

**Table 2:** Patient demographics

Category	Non Pregnant (*n* = 32)	Pregnant (*n* = 44)
Type of Infertility	N (%)	N (%)
Primary Infertility	14 (43.75)	31 (70.45)
Secondary Infertility	15 (46.88)	13 (29.55)
No indication of Infertility	3 (9.38)	0 (0.00)
Etiology		
Low response	2 (6.25)	2 (4.55)
Age (37 or older)	18 (56.25)	20 (45.45)
Endometriosis	2 (6.25)	7 (15.91)
Non determinate	3 (9.38)	3 (6.82)
PCO	3 (9.38)	4 (9.09)
Intrauterine insemination Failure	1 (3.13)	0 (0.00)
Tubal factor	2 (6.25)	7 (15.91)
Poor oocyte quality	1 (3.13)	0 (0.00)
Fertilization failure	0 (0.00)	1 (2.27)

After oocyte stimulation and fertilization, two or three high-quality embryos were transferred per a patient (Table 3). About 57.9% of the women achieved pregnancy. When the women were separated by the diagnosis of pregnancy, there was no difference between the non-pregnant group (average age: 39.8 ± 5.3) and pregnant group (average age: 38.3 ± 5.5) for number of embryos transferred, embryo stage or quality, and percent fragmentation at Day 3. This does suggest that any difference between women who did or did not become pregnant was not caused by the IVF procedure.

**Table 3:** Embryo’s demographics

Category	Non Pregnant	Pregnant
Number of embryos transferred (average)	2.69 ± 0.74	2.86 ± 0.52
Embryo stage (days)	3.34 ± 0.75	3.23 ± 0.61
Embryo quality (# cells at day 3)	7.29 ± 1.24	7.65 ± 0.96
Embryo quality (% fragmentation/day 3)	7.06 ± 4.81	5.03 ± 5.23

Value are mean ± standard deviation. Comparison between groups determined by the Student *t*-test. * *p* < 0.05. Non-significant differences were found between groups

### Collection and characterization of the endometrial tissue

**Fig. 1:**
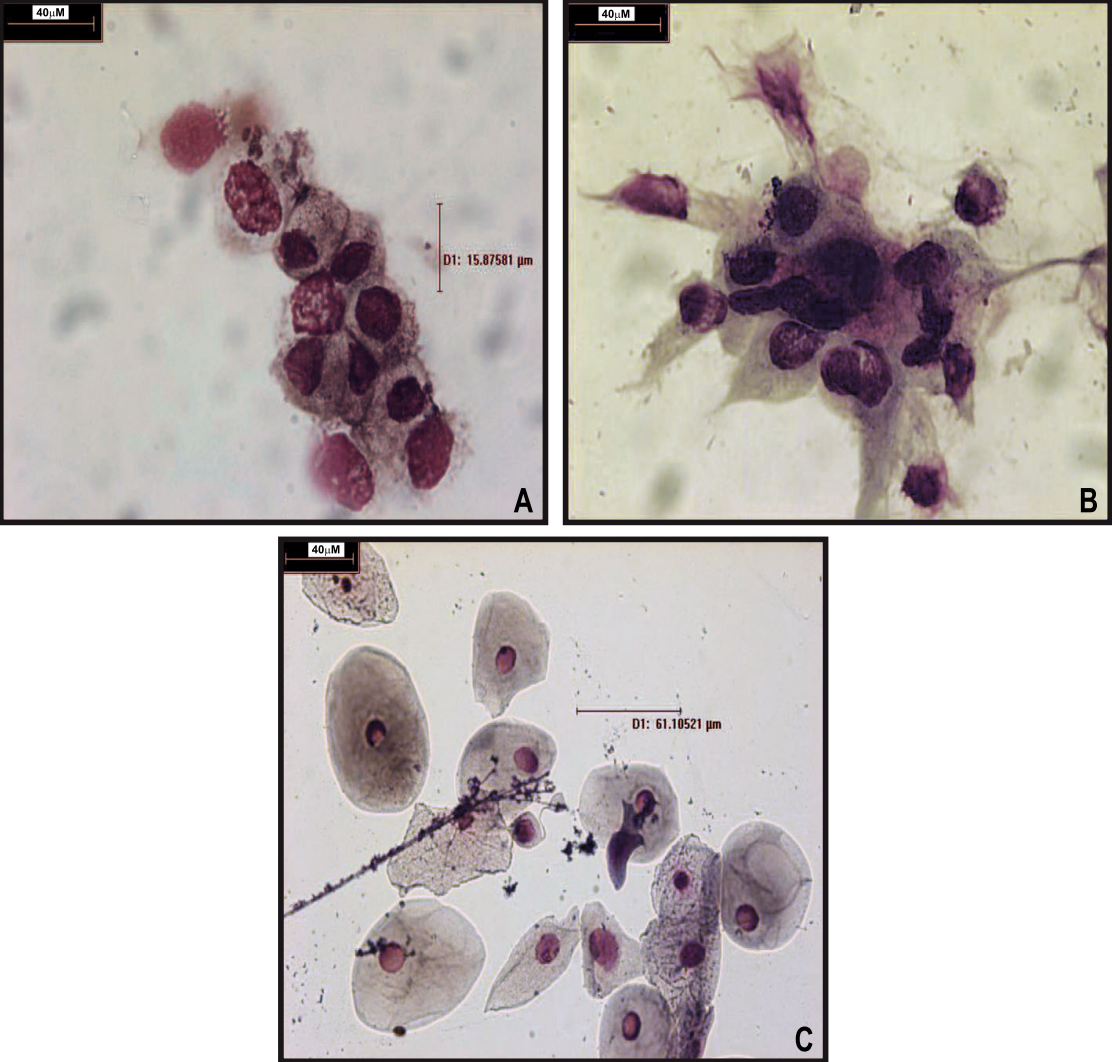
Endometrial cells attached to the cannula during embryo transfer**. a** and **b)** Cells that attached to the cannula were stained the hematoxylin and eosin, then visualized with an Olympus BX50 microscope (1000×). Their approximate size was 5–20 μm. **c** **)** For comparison, epithelial cells from the cervix were collected and stained

After embryo implantation, the cannula was washed with PBS. The PBS/cell solution was assessed for the presence of endometrial cells. The morphological evaluation, by H&E staining, suggests that the majority of cells were endometrial cells (Fig. 1a and b) and were not contaminated with cervical cells from the cervix neck (Fig. 1c). When the samples were assessed for endometrial markers, 85.53% of the samples were positive for HOXA-10 (65 positive samples). These results suggest the examined population were endometrial cells.

### Elevated CSF-1 and LIF gene expression is associated with pregnancy

**Fig. 2:**
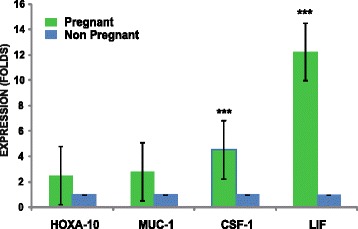
Transcriptional profile in endometrial cells with respect to pregnancy outcomes**.** Total RNA was collected from 76 women. The expression of HOXA-10, MUC-1, CSF-1, and LIF was determined by RT-qPCR. The fold differences in expression levels were calculated according to the 2^-ΔΔCt^ method, in duplicates. The data are expressed as mean ± standard error. Student’s *t*-test (Sigma Plot 12 Software) determined CSF-1 and LIF were significantly higher in endometrial cells collect from women who achieved clinical pregnancy than those who did not (***p < 0.001)

Four genes were assessed that are associated with endometrial receptivity. When the sample was separated into women who achieved pregnancy and failed to achieve pregnancy, there was no difference in the fold increase expression of HOXA-10 and MUC1. Interestingly, CSF-1 and LIF were significantly higher in women who could get pregnant (4.55 and 12.25-fold, respectively, *p* < 0.01, Fig. 2).

## Discussion

For more than 60 years, histological evaluations have been regarded as the standard for clinical diagnosis of endometrial abnormalities [17]. However, the accuracy, practicality, and functional relevance of this method as a predictor of endometrial receptivity remains a questionable [2]. In addition, it has been considered that the parameters of the ultrasound systems used to predict endometrial receptivity are still deficient in terms of specificity [18]. Thus, there is a need to find methods that can supplement ultrasound technology for determining endometrial receptivity in infertile patients.

Temporal and regional gene expression variations found within the endometrium make selecting ideal conditions for embryo implantation difficult. Serum analysis gives an overall average of not only the endometrium, but all tissues, which can significantly diminish specific signals. Thus, local assessment of the embryo implantation site should provide a more realistic evaluation of the endometrium receptivity. Therefore, cells that come in contact with a cannula specifically positioned on the surface of the endometrium could provide insight to the level of endometrium receptivity. Indeed, with the cells attached to the cannula, we were able to assess for genes associated with endometrial receptivity and their expression was associated with increased probability of pregnancy. This procedure allowed for current cycle analysis, without an additional invasive procedure. Moreover, due to the nature of IVF procedures, this sample was taken without altering the endometrium function, without affecting the embryo transfer, and with minimal contamination from other cells that came in contact with the cannula. This procedure could therefore prove to be a reliable method for the detection of genes to assess endometrial receptivity.

Numerous reports have demonstrated the importance of HOXA-10 on endometrium receptivity as well as implantation and pregnancy rates [19–22]. Recently, women with varying reasons of infertility were shown to have reduced HOXA-10 levels [22]. Here, our cohort was comprised of women suffering from infertility problems. We theorized that HOXA-10 expression might be lower in samples of patients that failed to achieve pregnancy. To the contrary, there was no significant difference in the HOXA-10 expression between the two groups. Similarly, a difference in the expression levels of MUC1 was expected, given that the association between MUC1 expression and the endometrial receptivity has been thoroughly investigated [23, 24] and MUC1 expression has been suggested as a key factor for embryo implantation. However, the lack of a difference in MUC1 expression between our two groups suggests that an alternative pathway may also play a role. Since pregnancy was achieved for some of the infertile women, this does posit that an uncharacterized alternative mechanism could promote implantation and pregnancy under certain HOXA10 and MUC1 expression conditions.

LIF has been consistently identified and associated with endometrial receptivity [2]. LIF regulates epithelial cell adhesive properties of the endometrium, affecting endometrial receptivity [25]. A recent report demonstrated that treatment with benzoic acid augments LIF expression and improve implantation rates [8]. Moreover, *Perilla frutescens* extracts [26] and calcitonin [27] were shown to induce LIF expression and increase implantation in an in vitro model. We postulated that for infertile women achieving pregnancy, LIF would likely be up-regulated. Indeed, we found that LIF was 12.25-fold higher among infertile women who achieved pregnancy, suggesting that augmenting LIF expression could promote implantation and pregnancy.

The role of CSF-1 during embryo implantation is still being deduced. Low CSF-1 serum levels were associated with recurrent miscarriages; CSF-1 deficient mice demonstrated decreased fertility [28]. The mechanism CSF-1 has on implantation rates remains elusive. However, one study suggests that CSF-1 is an important factor for placental function [7]. Women who achieved pregnancy could have augmented CSF-1 levels. Here, there was a 4.55-fold increase in CSF-1 expression for the infertile women who achieved pregnancy, suggesting that CSF-1 expression could improve IVF outcomes.

One key limitation of the study was the composition of the endometrium sample. The sample was collected during a standard IVF procedure, by normal contact with the endometrium with the cannula. Due to the nature of the endometrium, there was cellular transfer; however, the identity of the cells remains questionable. Of the possible cells that could be collected (luminal epithelium, glandular epithelium, stromal, etc.), Cullinan et al. demonstrated that the glandular epithelium cells are significant expressers of LIF. Nonetheless, other studies have since demonstrated that LIF can be expressed by the luminal epithelium cells [29–31]. A challenge we faced is the small amount of cells collected, which limits the potential endpoints that could be examined. Nevertheless, the goal of this study was to examine if the gene profile of the collected cells could aid in predicting pregnancy, and thus the cells’ identities are outside the scope of the study. Future studies are currently underway to determine the cells’ identities and the sample cellular composition by immunocytochemistry and in situ hybridization.

Another limitation to consider is the usefulness of the technique. Here, we are collecting the cells at the time of implantation and then assessing the gene profile. This would imply that if an unfavorable profile were determined that it would be too late. However, the purpose of this study was to determine the gene profile at implantation that would have a greater potential of leading to pregnancy. Once an optimal profile is codified, then pre-implantation samples can be collected and assessed. To that end, we are currently optimizing the procedure and are hopeful to apply the procedure between two to six hours before implantation.

In conclusion, our study provides a gene profile associated with endometrial receptivity in infertile women. Furthermore, we demonstrate a method that can be used to take a sample of the endometrium, which is minimally invasive and does not affect embryo transfer. Lastly, this method does give a local gene expression profile of the endometrium during the same cycle and could make possible to decide whether IVF treatments should be modified, giving a better chance of implantation and pregnancy.

## Abbreviations

CSF-1: Colony Stimulating Factor 1
HOXA: Homeobox A10
IVF: In vitro fertilization
LIF: Leukemia Inhibitory Factor
MUC: Mucin 1
PBS: Phosphate-buffered saline
